# Are there benefits of social overinclusion? Behavioral and ERP effects in the Cyberball paradigm

**DOI:** 10.3389/fnhum.2014.00935

**Published:** 2014-11-19

**Authors:** Michael Niedeggen, Natia Sarauli, Santi Cacciola, Sarah Weschke

**Affiliations:** Department of Educational Science and Psychology, Experimental Psychology and Neuropsychology, Freie Universität BerlinBerlin, Germany

**Keywords:** ostracism, Cyberball, ERP, overinclusion, expectation, social reward

## Abstract

Social participation can be examined using the Cyberball paradigm, a virtual ball-tossing game. Reducing the involvement of the participant is supposed to activate a neural alarm system, and to threaten fundamental social needs. Our previous findings indicate that the latter process can be linked to an enhancement of the centro-parietal P3 amplitude, signaling a modulation of the subjective expectancy of involvement. A preceding more frontal ERP component, the P2, does not depend of the probability of involvement, but reflects the appraisal of social reward. In this experiment, we examined whether overinclusion of participants enhances the satisfaction of social needs, reduces the P3 amplitude correspondingly, and affects central reward processing. In the control condition, participants (*n* = 40) were included (two co-player, ball possession 33%), and overincluded (ball possession 46%) in the experimental condition. In a counterbalanced design, we also controlled for the order of conditions. As predicted, overinclusion increased the satisfaction of social needs, with exception of “self esteem”, and reduced the P3 amplitude. As for the frontal P2, overinclusion only enhanced the amplitudes if the less frequent involvement (condition: inclusion) was experienced previously. The behavioral and P3 data suggest that the feelings of social belonging, meaningful existence, and control are related to the subjective expectancy of social involvement, and can be described in terms of a linear continuum ranging from exclusion to overinclusion. In contrast, appraisal of social rewards does not depend on the probability of involvement.

## Introduction

Social participation appears to be essential for human beings, and is vital to mental and physical health (Cacioppo and Cacioppo, 2012). Research in the last decade was largely based on ostracism, the exclusion of an individual by others (Williams, 2007). Following the seminal model of Williams, social exclusion has an immediate impact on basic human needs, such as the feeling of social belonging, or self-esteem (Williams and Zadro, 2005). Consequently, negative mood and distress is evoked in ostracized subjects.

According to the need-threat model, this first reaction to social exclusion is reflexive and unavoidable. In contrast to perceived fairness in trading games (Moretti and Di Pellegrino, 2010), the emotional response to social exclusion does apparently not rely on the credibility of the experimental setting, and is even triggered if exclusion is overtly scripted (Zadro et al., 2004). Because of its sensitivity and reliability, it has been assumed that the elicitation of the social need threat relies on a precognitive warning system.

In most behavioral and neuroimaging studies, the Cyberball paradigm has been used to elicit social exclusion (Williams et al., 2000; Williams and Jarvis, 2006). In this computerized version of a “ball-tossing” game, the participant is represented as an avatar on a computer screen. Two other avatars putatively represent two other human co-players connected via internet. In the following catch play, a ball is thrown and caught by the avatars. If the participant receives the ball in about a third of a time, “inclusion” is provided. “Exclusion” implies that the two assumed co-players are throwing the ball back and forth. In order to measure the effect of exclusion, the NTQ (Need Threat Questionnaire; Williams et al., 2000) is usually applied subsequent to the Cyberball game. In contrast to inclusion, social exclusion reduces the satisfactory level of belonging (e.g., “I felt disconnected”), self-esteem (e.g., “I felt good about myself”), meaningful existence (e.g., “I felt invisible”), and control (e.g., “I felt powerful”). The increase in need threat is associated with an increase in negative mood (van Beest and Williams, 2006).

Despite the reliability of the Cyberball paradigm in behavioral studies, the neurocognitive signature of social exclusion has not been consistently replicated. Whereas the dorsal anterior cingulate cortex (dACC) was closely related to the perceived distress (Eisenberger and Lieberman, 2004), additional regions of functional relevance were identified in a recent meta-analysis of the imaging data available (Cacioppo et al., 2013). However, these structures (left ACC, anterior insula) are rather related to emotional craving (Fisher et al., 2010), or uncertainty of decision making (Grinband et al., 2006; Singer et al., 2009) than to physical pain.

A further question is related to the functional specification of the reflexive system activated by ostracism. Following the ostracism model (Williams, 2007), a neural alarm system detects the slightest hint of ostracism, and triggers the reflexive painful response—unmitigated by situational factors. This implies that the reflexive process is unidirectional, and is activated by exclusion only. This idea is apparently substantiated by a Cyberball study (van Beest and Williams, 2006): Here, overinclusion—when contrasted to inclusion—did neither modulate the fundamental needs (as measured by the NTQ) nor negative mood significantly. This pattern of results was replicated in a recent neuroimaging study, and supports the notion of an alarm system selectively triggered by exclusion (Kawamoto et al., 2012). However, the results contrast earlier findings indicating that the inclusionary status follows a linear function, extending from overinclusion to inclusion, partial, and total ostracism (Williams et al., 2000). The expression of the inclusionary status is not only defined by the degree of involvement, but also by the incentives attached to the social involvement (van Beest and Williams, 2006). Both findings support the notion that the central processing of social participation cannot be sufficiently explained in terms of a reflexive process.

As suggested by our previous ERP studies (Gutz et al., 2011; Weschke and Niedeggen, 2013), subjective expectancy is a crucial factor in the evaluation of social participation. We analyzed the ERP responses time-locked to the reception of the ball in the Cyberball game. An experimental block of inclusion (two co-players, 33% ball possession) was compared with partial exclusion (two co-players, 16% ball possession). The target event (ball possession) triggered an ERP response defined by two components: a transient negativity at about 200 ms (N2), and a longer-lasting positivity starting at about 250 ms (P3). The centro-parietal part of the P3 shares the characteristics of the P3 evoked in the Oddball paradigm (Donchin, 1981; Rosenfeld et al., 2005), and signals the subjective probability of a task-relevant event (here: ball reception). Accordingly, the P3 amplitude was significantly enhanced if the ball was received less frequently by the participant. In order to refer to the effect of target probability on P3 amplitude, we will use the term* P3 effect* in the following.

The P3 effect appears to be related to the threat of social needs as measured by the NTQ: The amplitude of the parietal P3 was found to be correlated with the perceived ostracism intensity (Gutz et al., 2011). In contrast, crucial changes in the experimental setup not affecting the NTQ score (i.e., physical presence of co-players, Weschke and Niedeggen, 2013) did also not affect the P3 complex. We therefore concluded that cognitive processes related to the P3 effect, such as context-updating or expectancy towards feedback (Donchin and Coles, 1988, 1991; Hajcak et al., 2005; Polich, 2007), also affect the retrospective questionnaire on social exclusion, the NTQ.

Moreover, we also showed that ERPs can reveal additional cognitive processes related to the evaluation of social exclusion—not measured by means of the NTQ. As mentioned above, physical presence of the excluding co-players neither affected the NTQ nor the P3 effect (Weschke and Niedeggen, 2013). However, physical presence—as compared to the simulation of interaction via internet—enhanced an additional early frontal positivity (P2). This component has been elicited in numerous experimental studies in visual search (Luck and Hillyard, 1994), and memory recognition tasks (Evans and Federmeier, 2007), and was related to feature detection and cognitive matching processes. Moreover, the P2 has also been evoked in studies requiring reward prediction (Potts et al., 2006; Holroyd et al., 2011), and appears to signal the processing and appraisal of unexpected reward signals. Following this idea, ball reception in the Cyberball design might serve as a social reward, and changes in the amplitude of the P2 will therefore signal a modulation of the reward value (Weschke and Niedeggen, 2013).

In the present study, we used the Cyberball-ERP setup to examine the processing of overinclusion in the ball-tossing game. If the need threat system is activated exclusively in case of exclusion (Williams, 2007), overinclusion compared to inclusion should not affect the four fundamental needs (belonging, self-esteem, meaningful existence, and control; see for example Williams and Zadro, 2005). On the other hand, if overinclusion leads to an enhancement of social need satisfaction, the idea of a continuum defining the inclusionary state is supported (Williams et al., 2000). Since the Cyberball design shares the functional properties of the oddball paradigm, overinclusion should also affect the P3 effect. The inverse relationship between P3 amplitude and target probability was already demonstrated in previous oddball studies (Duncan-Johnson and Donchin, 1977; Polich and Margala, 1997). Our analysis did also focus on the P2 range providing insight on the evaluation of the reward (here: ball reception). A modulation of the P2 amplitude will indicate whether the social reward (ball reception) is enhanced or devaluated in case of overinclusion.

The evaluation of social inclusion is probably not only determined by the actual involvement in the game, but also by the previous experience. Transfer effects have recently been reported in two Cyberball experiments (Gutz et al., 2011; Tang and Richardson, 2013): In one study, negative mood was expressed more if social exclusion followed the previous experience of inclusion (Gutz et al., 2011), whereas an ameliorative benefit of inclusion following exclusion was reported in a second one (Tang and Richardson, 2013). We assume that these kinds of “transfer effects” reflect that experienced social participation modulates the expectations for future involvement. If these expectations are not fulfilled, a (re-)construction of a subjective probability model is necessary. Since an asymmetry of the adjustment process was found in our previous work (Gutz et al., 2011), we analyzed whether the behavioral (NTQ) and electrophysiological (P2, P3) effects are differently expressed if overinclusion preceded or followed inclusion.

## Materials and methods

### Participants

The procedure was approved by the local ethics committee at the FU Berlin. Fifty healthy subjects participated in the experiment. Due to a high number of artifacts in the EEG, eight participants had to be excluded. Additionally, two subjects had to be excluded because of missing data in the questionnaire, leaving a total of 40 for analyses. The participants had self-reportedly no history of psychiatric or neurological disorders and were not taking medication affecting the central nervous system. They were recruited in the university environment and gave their written consent for participating according to the Declaration of Helsinki. The subjects were randomly assigned to one of the experimental groups (*Inclusion first* *n* = 20, 11 female, mean age = 25.4 years; *Overinclusion first* *n* = 20, 12 female, mean age = 24.1 years). Since a cover story was required to induce the experimental effect, participants were informed about the experimental technique and aiming of the study afterwards. Participants got credit points for their studies.

### Task and design

The experimental setup (Cyberball game including the instructions) was programmed in MATLAB (R2012a, The MathWorks, Inc.). The program also provided the triggers for EEG recording. All participants were told that they took part in a study testing visual imagination capabilities. To keep up this cover story, the subjects first completed a short questionnaire about visual imagination ability (Vividness of Visual Imagery Questionnaire; Marks, 1973).

**Figure 1:**
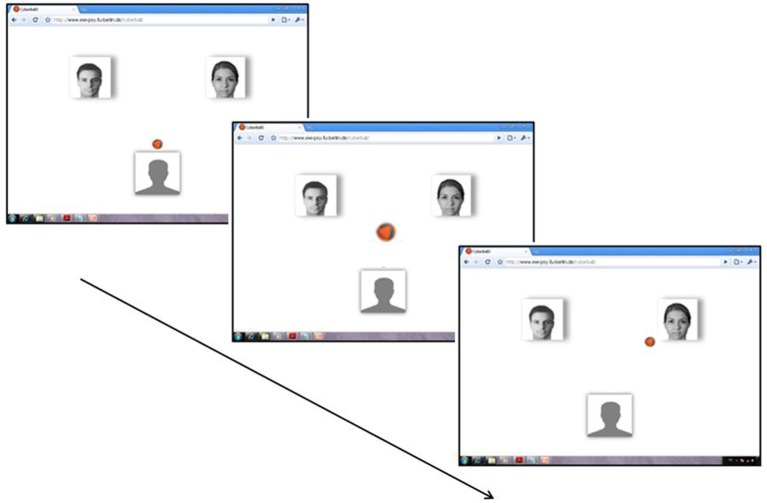
**Cyberball-ERP design: An internet display was imitated on the computer screen including the photos of two ostensible connected co-players**. The participant passed the ball to a co-player by pressing a corresponding button. Then, the ball appeared on a central position for 500 ms, and finally appeared next to the photo of the co-player. Ball possession of a co-player followed a computer-generated random time interval varying from 400–1.400 ms. The photographs of co-players depicted—and used in the experiment for all participants—refer to morphs of portraits taken from different persons. (For the setup of the screen: see Weschke and Niedeggen, 2013).

The setup of the Cyberball design followed an established design (see Gutz et al., 2011): Participants were told that they would play a ball-tossing game with two other co-players connected via internet (see Figure 1). For this reason, an internet display was simulated on the computer screen (7° × 7° at a viewing distance of 100 cm) displayed to the participants, including the photos of two putatively connected co-players. The participant could select the player to whom she/he wanted to throw the ball by pressing a corresponding button. To adapt to the technical requirements of ERP recording, animated avatars and a ball trajectory—included in the original Cyberball setup—were avoided: the ball was presented at a centered screen position for 500 ms, and then appeared next to the picture of a player. If one of the ostensible co-players received the ball, the temporal interval for the volley was randomly varied from 400–1.400 ms to enhance the belief of playing with humans.

Following the instructions and a short training introduction, all participants went through two blocks of the Cyberball game. Each block consisted of 200 ball throws and lasted about 7 min. In the experimental condition *Inclusion* (*INC*, proportionate ball possession), the participant received the ball in about one third of all ball throws (33%, approximately 66 times); in the experimental condition *Overinclusion* (*OI*; disproportionate ball possession), the probability of getting the ball was increased to 46% (approximately 92 times).

To account for the contrast effect assumed, the design comprised the between-subject “order of conditions”: 50% of the subjects started with the condition *Inclusion* (*INC first*) and the remaining 50% started with the condition *Overinclusion (OI first)*. The within-factors were defined by the probability of ball reception (“probability”: *INC* vs. *OI*), and the electrode positions (see below). As for the analysis of the ERPs, the analysis was focused on the ball reception of the participants.

After the second block, two NTQ questionnaires were handed out. The subjects were told to retrospectively fill out the questionnaires, the first one regarding the first block, and the second one regarding the second block. To make the separation of the two experimental blocks less difficult, one part of the ball-tossing game had to be imagined in the meadow and the other game on a beach. As already indicated in our previous studies (Gutz et al., 2011; Weschke and Niedeggen, 2013), the NTQ can reliably differentiate between the *Inclusion* and *Exclusion* condition, even if ratings on the first block are to be delivered with a temporal delay.

Beside of the NTQ, subjects had to estimate the probability of receiving the ball in the corresponding blocks. Moreover, the NTQ includes two scales measuring “negative mood” (e.g., “I felt angry”) and “perceived ostracism intensity” (e.g., “I was excluded”), which were extracted from the “Aversive Impact Index” (see for example Williams et al., 2000). Participants were also asked to rate the credibility of the cover story on a 5-point Likert scale (“My co-players were computer-generated.”).

After completing all questionnaires, the subjects were informed about the real aim of the study, and about the scripted behavior of the putative co-players.

### EEG recording and data analysis

#### EEG data

EEG data were recorded from 9 active electrode positions (frontal: F3, Fz, F4, central: C3, Cz, C4; parietal: P3, Pz, P4). Previous experiments had shown that these positions are highly sensitive to record the components of interest (Gutz et al., 2011; Weschke and Niedeggen, 2013). Ag/AgCl electrodes were embedded in an electrode cap (EASYCAP, Herrsching, Germany) mounted on the subjects’ head, and filled with electrode cream (Abralyt 2000, EASYCAP). Active electrodes (impedance < 5 kΩ) were referenced to linked earlobes (< 5 kΩ), with FCz serving as ground. Vertical and horizontal electrooculogram (EOG) were also recorded to control for ocular artifacts (< 20 kΩ). Biosignals were recorded continuously with a 40-channnel NuAmps amplifier (Software Acquire, Neuroscan Labs, Neurosoft, Inc., El Paso, TX). Data were band-pass filtered on-line (0.1–200 Hz) and sampled at 500 Hz. Off-line, EEG data were analyzed using “Brain Vision Analyzer” (Version 1.05, Brain Products GmbH, Gilching, Germany). EEG was segmented according to the onset of ball possession (−100–700 ms epoch length), filtered (0.3–30 Hz), and baseline corrected (−100–0 ms). Single EEG sweeps containing muscular or ocular artifacts were excluded from analysis, as well as EEG trials with high Alpha activity. Since there were more segments for ball possession in the condition *Overinclusion* by definition, the number of EEG segments was randomly chosen to adjust it to the number of segments obtained in the condition *Inclusion*. Averages and grand averages were calculated, separately for the two experimental groups (order of conditions), experimental conditions (probability), and nine active electrodes. Grand averages revealed three distinctive components: N2 (120–170 ms), an partially overlapping P2 (150–210 ms), and a sustained P3 complex with a parietal maximum extending from 320–400 ms. Mean amplitudes in these time windows were exported and analyzed using SPSS (version 19, IBM). Repeated measures ANOVAs were calculated including the between-subject factor “order of conditions” (*INC first* vs. *OI first*) and the within-subject factors “probability” (*INC* vs. *OI*), “electrode caudality” (frontal vs. central vs. parietal) and “electrode laterality” (left vs. central vs. right). Degrees of freedom and *p*-values were corrected according to Greenhouse-Geisser, if indicated, and corrected *p*-values will be reported in the following.

#### Behavioral data

For each participant, data of the NTQ and additional questions were read in SPSS (version 19, IBM) and NTQ scales were computed. The data were analyzed running a repeated measures ANOVA including the between-subject factor “order of conditions” (*INC first* vs. *OI first*) and the within-subject factor “probability” (*Inclusion* vs. *Overinclusion*). The four NTQ scales (belonging, self-esteem, control, meaningful existence) with a possible range of 1–5 were analyzed running a MANOVA. The data obtained for negative mood (e.g., “I felt sad”) (possible range: 4–20) and the perceived ostracism intensity (e.g., “I felt ostracized”) (possible range: 2–10) were analyzed in a separate ANOVA.

## Results

### Behavioral data

Behavioral data are presented in Table 1. The experimental manipulation of inclusion and overinclusion was reliably perceived by the participants: although the frequency of ball possession was slightly underestimated in both conditions (*INC*: 28.5%; *OI*: 44.9%), the difference between the mean estimated frequencies was significantly expressed (“probability”: *F*_(1,38)_ = 54.66, *p* < 0.001, *η*^2^ = 0.59). This main effect was not modulated by the between-factor “order of conditions” (*F*_(1,38)_ = 0.954, n.s.).

**Table 1:** **Behavioral results obtained in the two experimental conditions *Inclusion* (INC, ball possession 33%) and *Overinclusion* (OI, ball possession 46%)**.

	INC		OI	
GROUP	INC first	OI first	INC first	OI first
Estimated percentage of ball possession	29.90 (8.6)	27.20 (9.2)	48.40 (10.0)	41.4 (11.1)
Belonging	3.78 (0.62)	3.67 (1.04)	4.40 (0.47)	4.10 (0.63)
Self-esteem	3.52 (0.44)	3.15 (0.62)	3.33 (0.47)	3.33 (0.55)
Meaningful existence	4.47 (0.63)	4.05 (0.97)	4.72 (0.45)	4.65 (0.51)
Control	2.27 (0.72)	2.12 (0.77)	3.00 (0.70)	2.62 (0.90)
Negative mood	10.43 (1.70)	10.60 (1.78)	9.70 (1.71)	9.85 (1.56)
Perceived ostracism	4.20 (2.01)	4.75 (2.38)	2.45 (1.39)	2.45 (1.10)

*Mean values and standard deviations (in brackets) are provided for the two experimental groups (INC first: INC followed by OI; OI first: OI followed by INC)*.

The questionnaire also revealed that participants believed that the co-players were computer-generated (*INC*: *M* = 4.05, *SD* = 1.26; OI: *M* = 3.95, *SD* = 1.38). The credibility rating was comparable to the rating obtained in our previous study (Weschke and Niedeggen, 2013), and was not modulated by probability and group assignment.

Although a speeded response was not requested, participants’ reaction times were significantly decreased in the second block (first block: *M* = 660 ms, *SD* = 175, 8 ms; second block: *M* = 595 ms, *SD* = 154, 96 ms; *F*_(1,38)_ = 11.58, *p* = 0.002, *η*^2^ = 0.23). The effect on response time was not modulated by group assignment, and did therefore not depend on the probability of ball possession.

The four NTQ scales were also significantly affected by the frequency of ball possession (MANOVA: *F*_(4,35)_ = 5.47, *p* = 0.002, *η*^2^ = 0.39), although the temporal order of blocks did not elicit a significant interaction (*F*_(4,35)_ = 2.01,* p* = 0.190). In more detail, overinclusion led to a significant increase in the scales “belonging” (*F*_(1,38)_ = 14.01, *p* < 0.001, *η*^2^ = 0.28), “meaningful existence” (*F*_(1,38)_ = 11.49, *p* = 0.002, *η*^2^ = 0.23), and “control” (*F*_(1,38)_ = 18.18, *p* < 0.001, *η*^2^ = 0.32), but not “self-esteem”, (*F*_(1,38)_ = 0.00, n.s.). In none of the scales, temporal order of the blocks was found to modulate the effect of overinclusion (interaction with probability: “belonging” (*F*_(1,38)_ = 0.46, *p* = 0.50, *η*^2^ = 0.012), “meaningful existence” (*F*_(1,38)_ = 1.95, *p* = 0.17, *η*^2^ = 0.05), “control” (*F*_(1,38)_ = 0.65, *p* = 0.43, *η*^2^ = 0.02), “self-esteem”, (*F*_(1,38)_ = 3.30, *p* = 0.08, *η*^2^ = 0.08).

Two additional scales were reflecting the affective and cognitive evaluation of the social participation in the Cyberball game: negative mood was significantly decreased in overinclusion as compared to inclusion (*F*_(1,38)_ = 30.26, *p* < 0.001, *η*^2^ = 0.44), as well as the perceived ostracism intensity (*F*_(1,38)_ = 8.11, *p* = 0.007, *η*^2^ = 0.18). Again, temporal order did not modulate the effects significantly.

In none of the scales, a significant main effect of the between-factor “order of conditions” was found.

### ERP data

**Figure 2:**
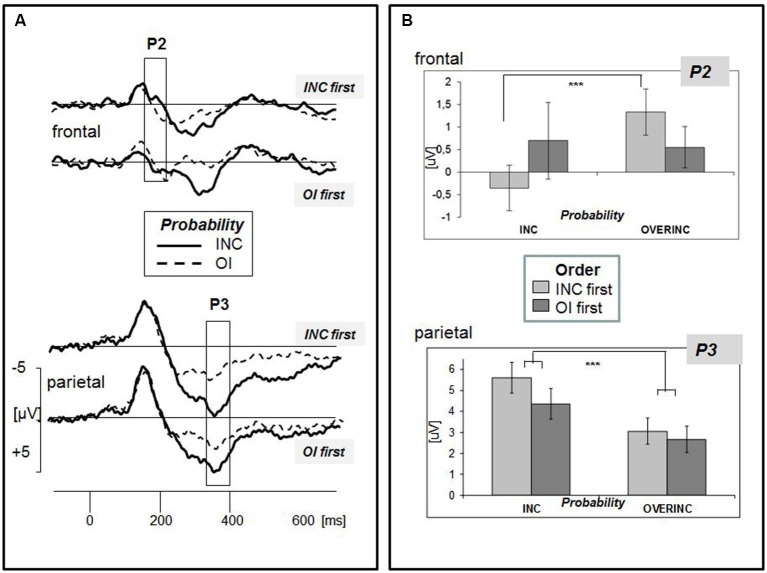
**(A)** Grand-averaged ERPs for the event “ball possession of the participant” separated for the two experimental groups (order of condition: “INC first” vs. “OI first”). Electrodes are pooled to the clusters “frontal” and “parietal”. Two experimental conditions related to different probabilities of ball possession are superimposed (INC: inclusion, 33%; OI: overinclusion, 46%). Amplitude differences between the conditions were observed in two time windows, 150–210 ms (P2, see frontal leads) and 320–400 ms (P3, see parietal leads). **(B)** Mean ERP amplitudes of the two components separated for the effect of experimental condition “probability” (inclusion vs. overinclusion) and “order of conditions” (inclusion first vs. overinclusion first). Bars indicate the standard error of mean.

The grand-averaged ERPs evoked by the event “ball possession” are depicted in Figure 2. Three time ranges were exported for further analyses: The N2 range (130–180 ms) was marked by a parietal negative peak at about 170 ms, and released by a marked frontal positivity mostly expressed in the condition “overinclusion” (P2, 150–210 ms). Replicating our previous findings (Gutz et al., 2011; Weschke and Niedeggen, 2013), ball reception triggered a late positive complex with a centro-parietal maximum extending from 320–400 ms (P3). The following analysis was focused on the P2 and P3. In line with our previous results, no effect on the N2 amplitude was induced by modulating the probability of ball reception.

#### P2 (150–210 ms)

According to the grand-averaged ERPs (see Figure 2A), the P2 amplitude appears to be more expressed in the condition “overinclusion”. The ANOVA confirmed a significant main effect of “probability” (*F*_(1,38)_ = 7.000, *p* = 0.012, *η*^2^ = 0.156). The effect of probability, however, was significantly modulated by the factor “caudality” and the order of experimental conditions (“probability” × “caudality” × “order of conditions”: *F*_(2,76)_ = 12.346, *p* < 0.001, *η*^2^ = 0.245). The interaction of the relevant two experimental factors (probability, order) was only confirmed at frontal leads (“order of conditions” × “probability”: *F*_(1,38)_ = 4.797, *p* = 0.035, *η*^2^ = 0.112), but not at central and parietal leads (each *p* > 0.1). As revealed in Figures 2A,B, the difference between the inclusionary states was only found to be significant for the experimental group of subjects starting with inclusion (*INC first*, “probability”: *F*_(1,19)_ = 21.065, *p* < 0.001, *η*^2^ = 0.526), but not for the group starting with overinclusion (*OI first*, “probability”: *F*_(1,19)_ = 1.724, *p* = 0.205). None of the effects aforementioned was modulated by the laterality of the electrodes.

#### P3 (320–400 ms)

The positive amplitude was more expressed in the inclusion condition as compared to overinclusion (Figure 2A). The effect of probability was differently expressed at frontal, central, and parietal electrode positions (“caudality” × “probability”: *F*_(2,37)_ = 11.857, *p* < 0.001, *η*^2^ = 0.391). Separate analyses for the three clusters showed that P3 effects were significantly expressed at central and parietal leads, with larger effects at the parietal (“probability”: *F*_(1,38)_ = 22.252, *p* < 0.001, *η*^2^ = 0.369) as compared to the central leads (“probability”:* F*_(1,38)_ = 15.252, *p* < 0.001, *η*^2^ = 0.286). Although mean amplitudes appeared to be more expressed in the group *INC first*, the between-subject factor “order of conditions” was not significant (*F*_(1,38)_ = 0.926, n.s.). Moreover, neither laterality nor temporal order did modulate the effect of probability.

## Discussion

### Summary of results

Overinclusion had a remarkable effect on the processing of social participation: Following the NTQ data, increasing the probability of ball possession significantly increased the scales “belonging”, “meaningful existence”, and “control”. Furthermore, negative mood was significantly decreased, as well as the perceived ostracism intensity. The behavioral data indicate an enhancement of social need satisfaction. These effects were associated with a corresponding decrease in the P3 amplitude. A similar effect can be obtained in the oddball paradigm, if target frequency is increased (Polich and Margala, 1997).

The temporal order of the experimental conditions “inclusion” and “overinclusion” did neither modulate the behavioral nor the P3 effect aforementioned. In contrast to our predictions, an asymmetry in the adjustment of subjective expectancy depending on the temporal order of the conditions was not found. However, the temporal order had a selective effect on the frontal P2, previously related to the appraisal of social reward signals (Weschke and Niedeggen, 2013). This component was significantly increased in case of overinclusion—given the previous experience of inclusion. A corresponding ERP effect was not obtained if overinclusion preceded inclusion.

These results will be discussed in detail.

### Behavioral effects of overinclusion

Behavioral data on the effect of overinclusion in Cyberball are not consistent. In a first internet-based study (Williams et al., 2000), the inclusionary status was varied in four degrees (complete exclusion, partial exclusion, inclusion, and overinclusion), and a significant relationship to the NTQ scales “belonging” and “self-esteem” was found. The linear effect also extends to the participants’ mood, and apparently supports the idea of an inclusionary continuum. The data obtained in two other studies (van Beest and Williams, 2006; Kawamoto et al., 2012) provided no evidence indicating that overinclusion affects self-reported social pain. They rather revealed that distinct neural networks are involved if participants are excluded, such as the dACC, or the right ventrolateral prefrontal cortex (Kawamoto et al., 2012). The latter is in line with the assumption that the neural alarm system is exclusively activated by ostracism, but not affected by overinclusion.

Our findings add some new aspects to the ongoing discussion: Summarizing the single NTQ scales, a significant effect of overinclusion indicated an enhancement of social need satisfaction. In line with the reduction of negative mood one might conclude that overinclusion is experienced more positive than inclusion. The findings are in contrast to the behavioral results aforementioned (van Beest and Williams, 2006; Kawamoto et al., 2012), and appear to be more compatible with the idea of an inclusionary continuum resembling the characteristics of a “sociometer”. The up- and down-regulation of need threat and mood—triggered by ball possession in the Cyberball game—are probably related to an internal gauge monitoring the degree of social acceptance (Leary et al., 1998). The lack of an effect of overinclusion in previous studies can rather be related to differences in the experimental setup: In one study (van Beest and Williams, 2006), the addition of financial incentives apparently affected the subjective well-being of the participants in the Cyberball setup. In another study (Kawamoto et al., 2012) multiple short blocks of inclusion, overinclusion, and exclusion were presented, and the subjects apparently had to fill out questionnaires between these blocks.

However, we have to consider the NTQ scale not modulated by overinclusion—self-esteem. Self-esteem has been closely attached to the perception of rejection in the sociometer model (Leary et al., 2001). This dissociation within the NTQ scales might signal that self-esteem is not an inner gauge of social acceptance, but a generalized indicator of interpersonal appeal (Blackhart et al., 2009). One might also speculate that some components related to a social need-threat model (Williams, 2007) are selectively affected by social rejection, but not by social acceptance. This is in line with neuroimaging studies: Structures in the ACC and prefrontal cortex are exclusively activated by exclusion, but not by overinclusion (Kawamoto et al., 2012).

### ERP correlates of overinclusion

As previously observed for the case of social exclusion induced in the Cyberball game (Gutz et al., 2011; Weschke and Niedeggen, 2013), overinclusion affected the P3 effect: The P3 amplitude was significantly reduced if the involvement in the ball-tossing game was increased. The effect corresponds to the participants’ estimation of ball possession (see Table 1), and was—in contrast to the preceding P2—not modulated by the order of the experimental conditions.

With respect to the frequency of the target event (ball possession), the P3 effect observed in Cyberball shares the characteristics of the oddball-triggered P3 complex related to controlled processing, such as context updating (Donchin, 1981) and the modulation of subjective probabilities (Duncan-Johnson and Donchin, 1977). In case of social exclusion, the significant increase of P3 amplitude was supposed to reflect the re-adjustment of subjective expectancy of social participation (Gutz et al., 2011; Weschke and Niedeggen, 2013). In an exclusionary rally, the enhancement of the P3 effect triggered by casual ball receptions can be related to the processing of unexpected feedback (Hajcak et al., 2005). Increasing the frequency of the task-relevant event correspondingly decreases the amplitude of the P3 amplitude (Polich and Margala, 1997), and this effect applies for the increase in ball possession in overinclusion, too.

We have previously proposed that the expectancy of getting included in the Cyberball game is reflected in the P3 effect, and affects the responses in the retrospective questionnaire, NTQ (Weschke and Niedeggen, 2013). Our recent data indicate that this relation holds for most of the NTQ scales (“belonging”, “meaningful existence”, and “control”), but not for “self-esteem”. We already proposed in a previous study (Weschke and Niedeggen, 2013) that the NTQ scales—related to the feeling of social belonging and control in social interaction—provide a retrospective measure of the status of social acceptance, and are determined by the subjective probability of involvement. For these scales, P3 can serve as an online-measurement of systematic variation. State self-esteem is obviously less dependent on subjective expectancies in social interactions, and is apparently closely related to the direct experience of social rejection.

Although subjective expectancy apparently affects both, NTQ scales and the P3 amplitude, significant correlations of questionnaire and electrophysiological data were not obtained. The lack of predictive power in single participants is probably due to the temporal lag between the experience of social participation and its rating in the questionnaire. Please note that both questionnaires, the one referring to inclusion and the one referring to overinclusion, were to be answered following the second block.

### Transfer effects

In contrast to earlier findings based on the transition from inclusion to exclusion (Buckley et al., 2004; Gutz et al., 2011; Tang and Richardson, 2013), we found no evidence in the questionnaire data for a transfer effect between inclusion and overinclusion in the behavioral data. Previous experience of overinclusion (group: *OI first*) did not enhance the expectation of further social inclusion, and social needs are obviously not threatened by the—relative—decrease in ball possession in the second block. An asymmetry in adjustment processes was also not obtained in our online-measurement, the P3 effect.

The lack of a transfer effect probably indicates that the re-construction of subjective probabilities of social involvement is a rather fast process. This corresponds to earlier findings obtained in the oddball paradigm (Lindín et al., 2004) providing evidence that adjustment to a low target probability required less than 100 trials.

However, an asymmetry in the order of experimental conditions was obtained for the frontal P2. Its amplitude was significantly increased in case of overinclusion, given that inclusion was experienced previously. If overinclusion preceded inclusion, no such effect was found. A corresponding ERP reflection with comparable latency and topography has been also identified in a previous Cyberball study (Weschke and Niedeggen, 2013), and its amplitude was modulated by the physical presence of the co-players in an exclusion condition as compared to the—putative—internet connection. In line with studies on reward prediction (Potts et al., 2006; Holroyd et al., 2011), the P2 was related to the processing of an unpredicted—here: social—reward signal. The actual study points out that overinclusion in the Cyberball game does not provide a higher social reward *per se*, but depends on the preceding experience of the participant.

The differential effects on P2 and P3 amplitudes also emphasize that multiple affective and cognitive mechanisms contribute to the central processing of social exclusion elicited in the Cyberball paradigm.

## Conclusion

In case of overinclusion in the Cyberball game, the modulation of feelings of social belonging and control is in line with the idea of an inclusionary continuum. The retrospective evaluation is related to the participants’ expectancy on involvement—as reflected in the P3 effect. Expectancy, however, is not related to the participant’s self-esteem which appears to be more sensitive to ostracism. Finally, the ERP data reveal that the evaluation of social reward signals in Cyberball depend on previous experience.

## Conflict of interest statement

The authors declare that the research was conducted in the absence of any commercial or financial relationships that could be construed as a potential conflict of interest.
